# Supplementary material for: Activation of Prp28 ATPase by phosphorylated Npl3 at a critical step of spliceosome remodeling
Source: Nat Commun. 2021 May 25;12:3082. doi: 10.1038/s41467-021-23459-4 (PMC8149812; doi:10.1038/s41467-021-23459-4)
Supplement: Supplementary file 1 — Supplementary Information [file 41467_2021_23459_MOESM1_ESM.pdf]

Activation of Prp28 ATPase by Phosphorylated Npl3 at a Critical Step of Spliceosome Remodeling

Yeh et al.

Supplementary information

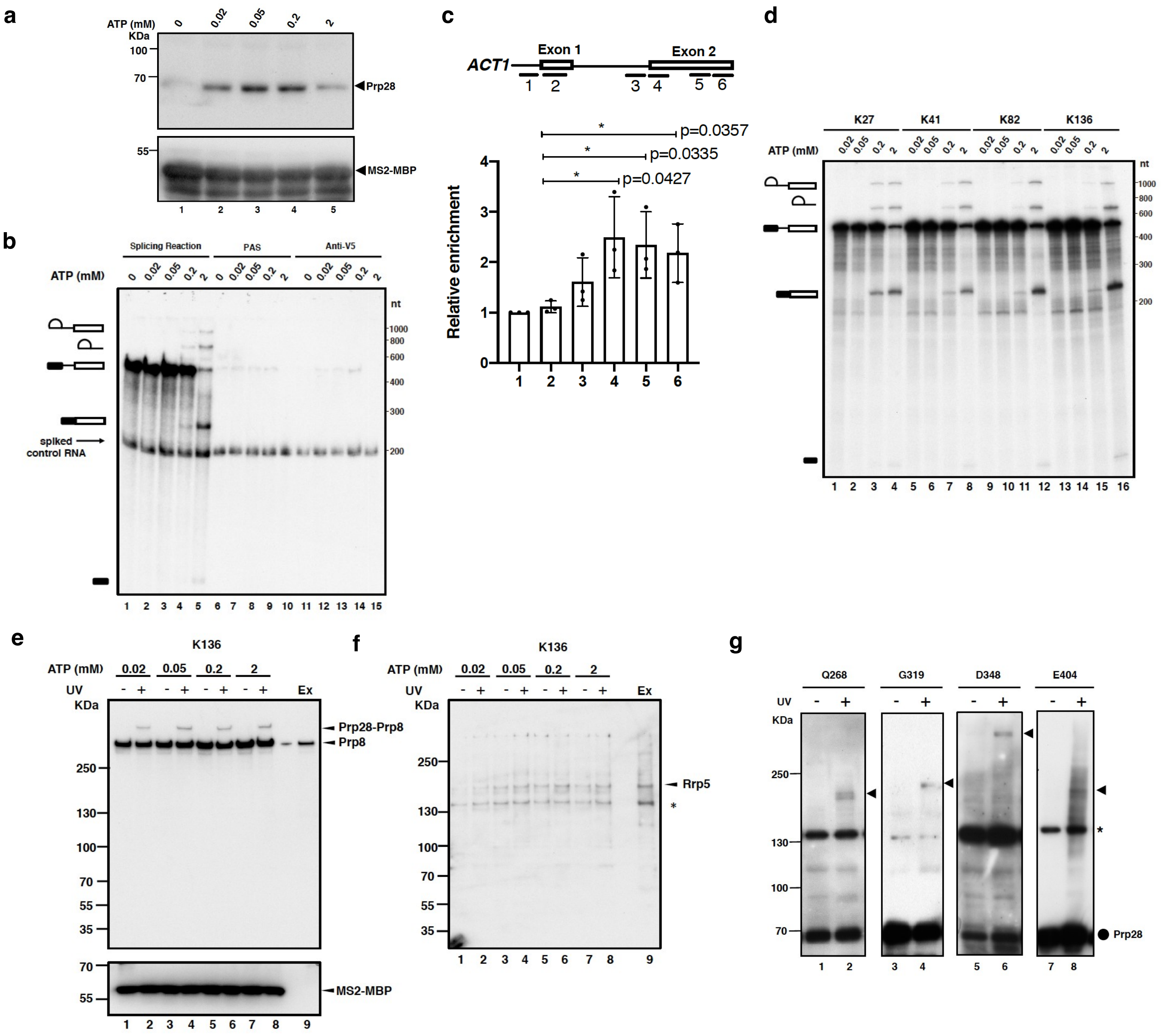

Supplementary Figure 1: Prp28 contacts key proteins at the heart of spliceosome. a, Prp28 transiently associates with the spliceosome during splicing. Splicing extracts containing V5-tagged Prp28 were used to assemble spliceosome on MS2-stem-loop-tagged *ACT1* pre-mRNA at various ATP concentrations. After pulling down the spliceosome, the presence of Prp28-V5 and MS2-MBP were visualized by immunoblotting using anti-V5 and anti-MBP antibody, respectively. **b,** Specificity of the Anti-V5 antibody. Splicing reactions (lanes 1–5) were done in none-tagged Prp28 extracts at 0, 0.02, 0.05, 0.2, or 2 mM ATP and a portion was subjected to immunoprecipitation without antibody (PAS; lanes 6–10) or with anti-V5 antibody (lanes 11–15). Relative loadings are 1:10 for splicing reactions alone (lanes 1 to 5) vs. immunoprecipitated reactions (lanes 6 to 15). A 200-nt spiked-in control RNA as indicated. nt, RNA size markers. **c,** ChIP analyses showed that Prp28's association with the spliceosome. Data are presented as mean values +/- SEM, n = 3 (biological repeats), *p < 0.05, the exact p value is as indicated; unpaired two-tailed t-test. **d,** Extracts made from Prp28-K27, -K41, -K82, or -K136 strains are active in splicing. **e-f,** Validation of mass spectrometry identification of Prp8. The crosslinked species of prp28- K136 was identified by anti-Prp8 (lanes 1–8). **f,** The same blot was stripped and re-probed by anti-Rrp5 (Rrp5 serves as a negative control). Ex, K136 splicing extracts (lane 9). Detection of MS2-MBP serves as a loading control (bottom panel in **e**). **g,** BPA-mediated crosslinking experiments using extracts made from Prp28-Q268, -G319, -D348, and -E404 strains yielded unidentified crosslinked species. Asterisk, nonspecific background band. All above experiments were repeated three times with similar results. Source data are provided as a Source Data file.

a

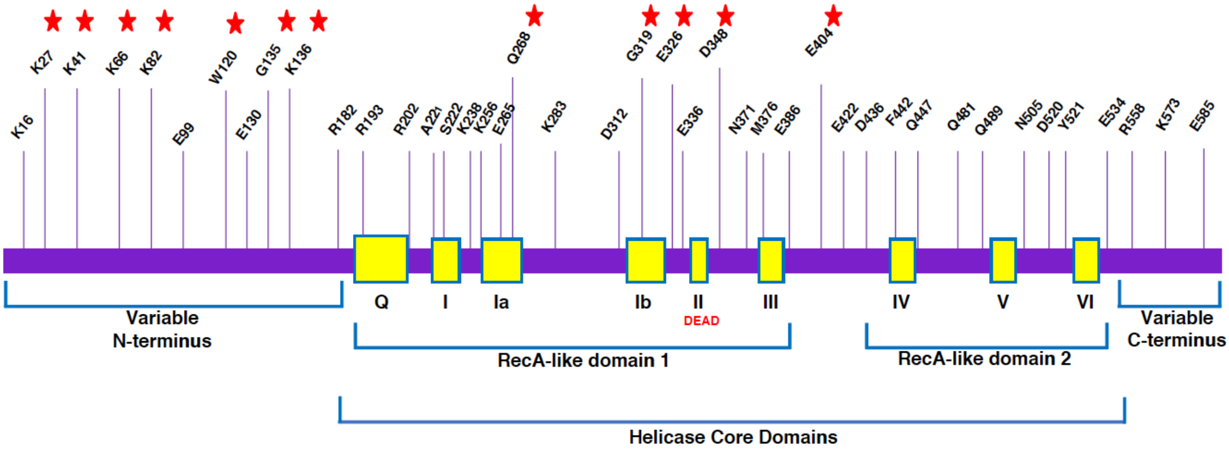

b

```
                                                                 
S.cerevisiae 183 DLLRVIIQELRFPSPTPIQRITIPNVCNMKQYRDFLGVASTGSGKTLAFVIPILIKMSRS
H.Sapiens    402 ILE--VIDKCGYKEPTPIQRQAIPIGL---QNRDIIGVAETGSGKTAAFLIPLLVWITTL
                  *    *       ******  **      * **  *** ****** ** ** *

                                          268
S.cerevisiae 243 PPRPPSLKIIDGPKALILAPTRELVQQIQKETQKVTKIWSKESNYDCKVISIVGGHSLEE
H.Sapiens    457 PKIDRIEESDQGPYAIILAPTRELAQQIEEETIKFGKP------LGIRTVAVIGGISRED
                 *          ** * ******** ***  ** *  *                ** * *
                                          482

                                 319    326                  348
S.cerevisiae 303 ISFSLSEGCDILVATPGRLIDSLENHLLVMKQVETLVLDEADKMIDLGFEDQVTNIL---
H.Sapiens    511 QGFRLRMGCEIVIATPGRLIDVLENRYLVLSRCTYVVLDEADRMIDMGFEPDVQKILEHM
                   * *  ** *  ******** ***  **       ****** *** ***  *  **
                                 527    534                  556

S.cerevisiae 360 ------TKVDINADSAVN-----------RQTLMFTATMTPVIEKIAAGYMQKPVYATIG
H.Sapiens    571 PVSNQKPDTDEAEDPEKMLANFESGKHKYRQTVMFTATMPPAVERLARSYLRRPAVVYIG
                          *   *               *** ****** *  *  *  *   *    **

                  404
S.cerevisiae 403 VETGSEPLIQQVVEYADNDEDKFKKLKPIVAKYDPPIIIFINYKQTADWLAEKFQKETNM
H.Sapiens    631 SAGKPHERVEQKVFLMSESEKRKKLLAILEQGFDPPIIIFVNQKKGCDVLAKSLEK-MGY
                           * *      *   * *       ******* * *   * **    *
                  632
```

c

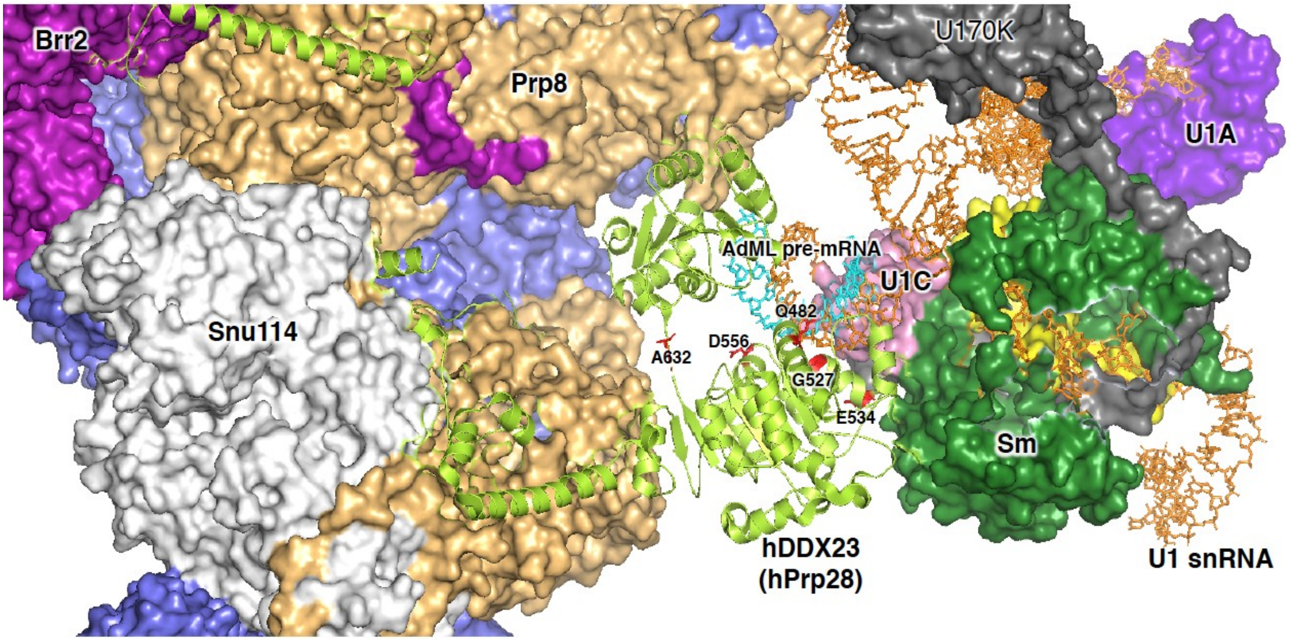

Supplementary Figure 2: Positions of BPA-replaced amino acids in yPrp28 that yielded crosslinked species. a, Schematic representation, not to scale, showing domains and motifs of yPrp28 common to the DEAD-box family. The catalytic helicase core is composed of two RecA-like domains (domain 1 and domain 2), which contain conserved motifs. The variable N-and the C-terminus regions are also indicated. A total of 42 amino-acid residues in Prp28 were individually replaced by BPA for crosslinking experiments. The 12 red-star-marked positions are capable of crosslinking to various proteins. **b,** Sequence alignment of Prp28 from *S. cerevisiae* and *H. sapiens*. Conserved residues within the first RecA domain were highlighted in grey and the BPA- replaced amino-acids are highlighted turquoise. **c,** A PyMol representation of the partial Cryo-EM structure of the human pre-B complex (Protein Data Bank 6QX9). The marked amino acids (e.g., Q482) are residues in the human Prp28 corresponding to residues of the yeast Prp28 highlighted in turquoise in **b**. The Cryo-EM structure of the labeled human pre-B complex is displayed by PyMol 2.3.2 and the saved file is provided as the Supplementary Software 1. Source data are provided as a Source Data file.

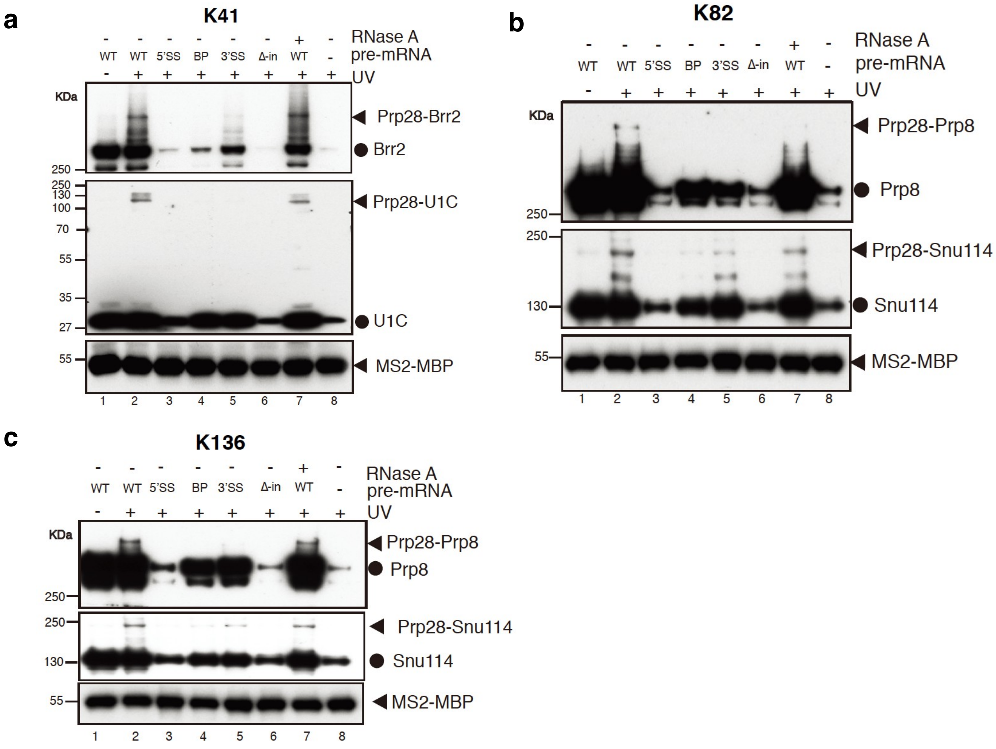

Supplementary Figure 3: Crosslinks of Prp28^{BPA} to Brr2, U1C, Prp8, and Snu114 are splicing dependent. **a,** Prp28-K41^{BPA}, **b,** Prp28-K82^{BPA}, and **c,** Prp28-K136^{BPA}. WT, wild-type transcript; 5'SS, 5'SS mutation (GUAUGU to AUUUGU); BP, branch-site A-to-C mutation (UACUAAC to UACUACC); 3'SS, 3'SS (AG | AG into AC | AC, where | indicates the 3' splice site); Δ-in, intron-less transcript. The transcript we used in the reaction contains a well-known "pseudo-branch-site", UACUAAG, which marginally functions as a branch site, upon losing the genuine wild-type branch site (UACUAAC). Therefore, a minor fraction of spliceosome might have been assembled on the branch-site mutant transcript, offering a plausible explanation for the weak crosslinking of Snu114 to Prp28. In addition, it is well known that the 3'SS-mutated transcript (i.e., the so-called ACAC transcript) still allows spliceosome to assemble for completing the first splicing step. This may explain, once again, the weak crosslinking of Snu114 and Prp28. All above experiments were repeated three times with similar results. Source data are provided as a Source Data file.

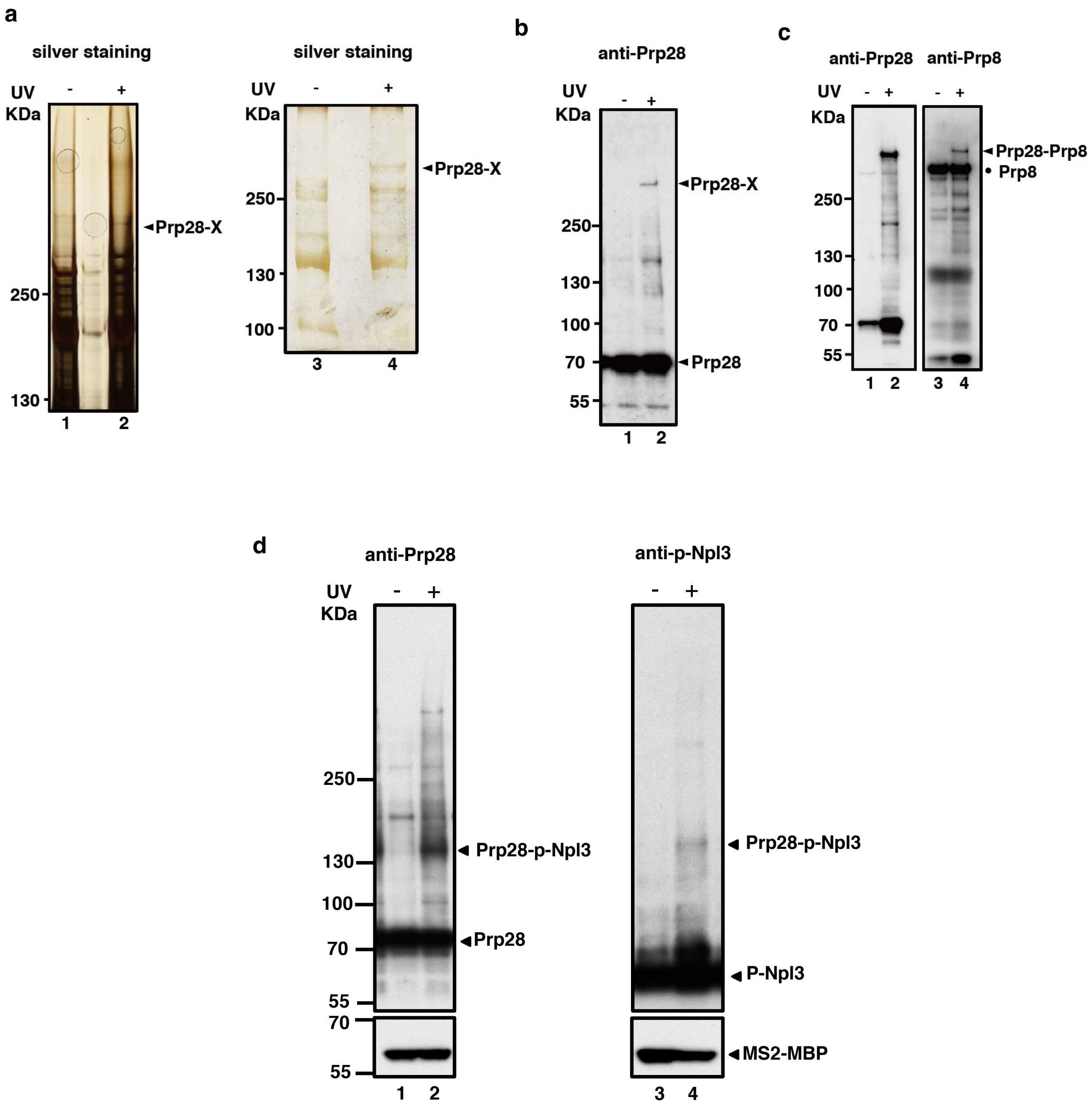

Supplementary Figure 4: Identification of the protein crosslinked to Prp28-K136^{BPA}. **a,** Two scale-up (100X) crosslinking reactions were done without (-) or with (+) UV irradiation. Pulled-down materials were then separated by 3–8% gradient SDS-PAGE and silver stained (left panel, lanes 1, 2). The replicate experiment was done (right panel, lanes 3 and 4). Bands at this location (Prp28-X) from both lanes (-UV, +UV) were excised and then subjected to mass spectrometry analysis separately. **b,** The location of the crosslinked species were then confirmed by immunoblotting. **c**, Validation of mass-spectrometry prediction of Prp8 as the crosslinked protein. The left panel was probed with anti-Prp28 antibody and right with anti-Prp8 antibody. **d,** Validation of mass-spectrometry prediction of Npl3 as the crosslinked protein. The left panel was probed with ant-Prp28 antibody and the right panel with anti-p-Npl3 antibody. All above experiments were repeated three times with similar results. Source data are provided as a Source Data file.

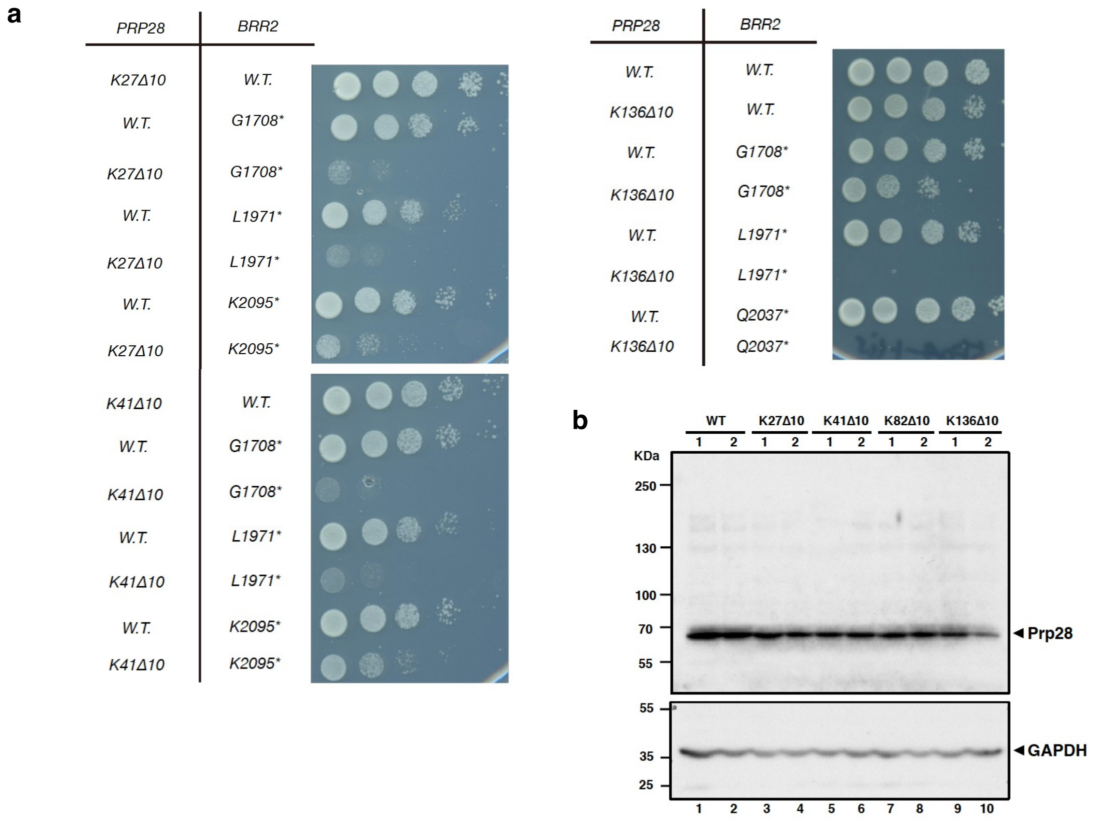

Supplementary Figure 5: Genetic interactions between Prp28 and Brr2. **a,** Yeast strains harboring various combinations of *prp28* and *brr2* alleles spotted in a dilution series and grown on YPD medium at 30˚C. Details of the *brr2* alleles tested are described in Supplementary Table 4. **b,** The presence of Prp28 in the wild-type and the "Δ10"-deletion strains were visualized by immunoblotting using anti-Prp28 antibody. Detection of GAPDH was used as an internal control. Two biological repeats were done. Source data are provided in the Source Data file.

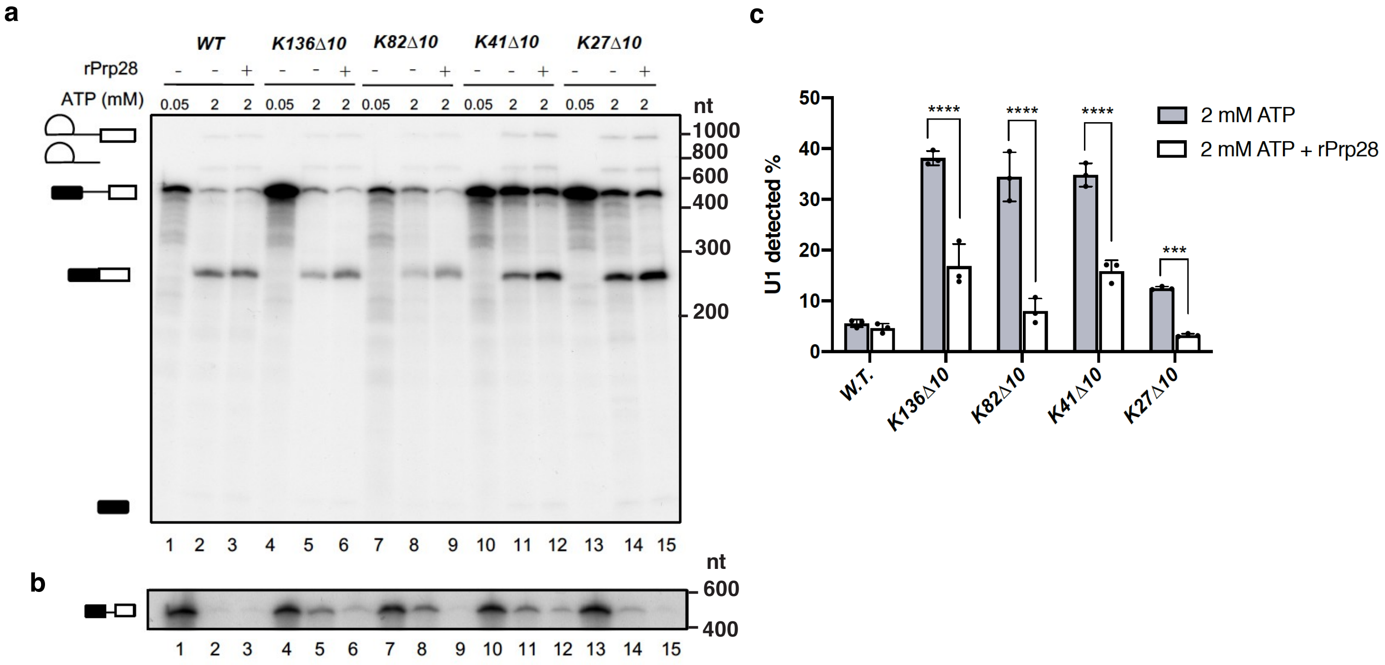

Supplementary Figure 6: Accumulation of U1 snRNP in *prp28-K27Δ10*, *prp28-K41Δ10*, *prp28-K82Δ10*, and *prp28-K136Δ10* splicing reactions. a, Splicing reactions were carried out at 0.05 mM (lanes 1, 4, 7, 10, 13) and 2 mM (lanes 2, 5, 8, 11, 14) of ATP with (+) or without (-) addition of recombinant Prp28 at 2 mM of ATP (lanes 3, 6, 9, 12, 15). The experiment was repeated three times with similar results. **b,** Reaction mixtures were immunoprecipitated with anti- Prp40 antibody to probe for the presence of U1 snRNP. The experiment was repeated three times with similar results. **c,** Quantification of retained U1 snRNP from **(b).** The Y axis represents the amount of U1 snRNP detected from the spliceosome. The amount of U1 snRNP detected in the wild-type reaction at 0.05 mM ATP is set to 100% for data normalization. Amounts of RNA from **(b)** were quantified by a PhosphoImager. Data represent means ± SEM, n = 3 biological repeats, ***P<0.001(P value=0.001), ****P<0.0001. Statistical significance was determined by unpaired two- tailed t-test. nt, RNA marker nucleotides. Source data are provided as a Source Data file.

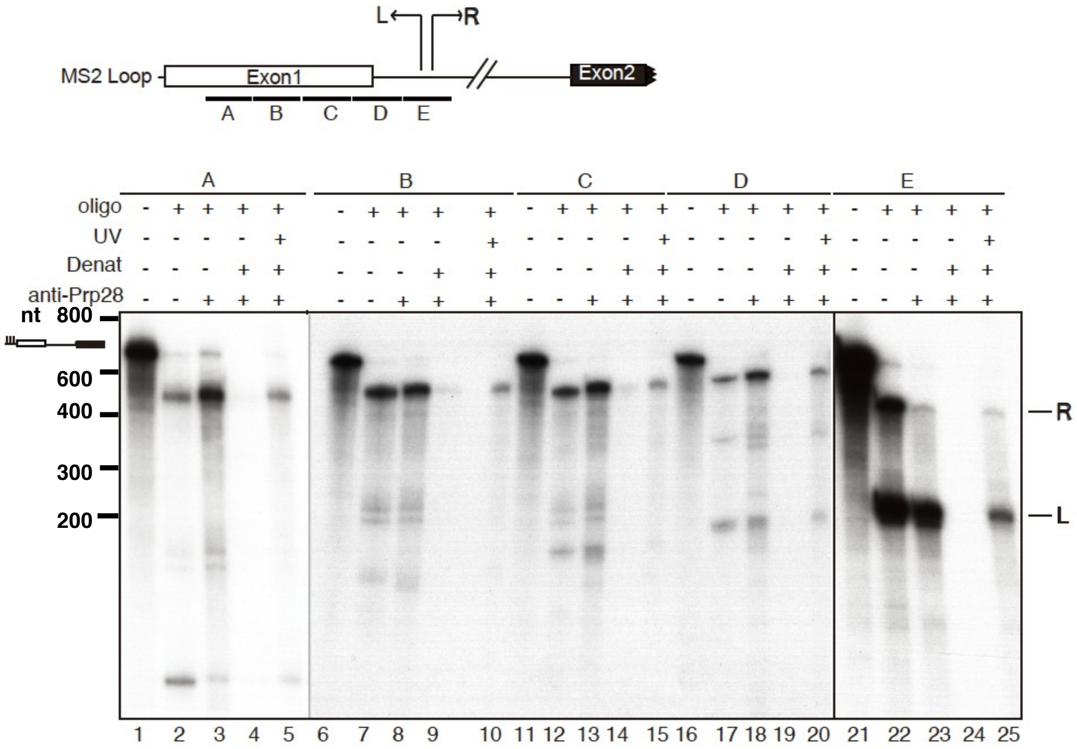

Supplementary Figure 7: RNase H mapping of Prp28 crosslinked region on *ACT1* pre-mRNA. The top diagram depicts oligonucleotides (A, B, C, D, and E) used to direct RNase H digestion on MS2 stem-loop-tagged radiolabeled *ACT1* transcript (10-fold specific activity). Open box, Exon 1; solid box, Exon 2; solid line, intron. Spliceosome was first assembled on transcript in five independent splicing reactions. Oligonucleotides were then added for RNase H digestion, which was followed by 254-nm UV irradiation. After complete denaturation of the reactions, anti-Prp28 antibody was used to immunoprecipitate Prp28-crosslinked RNAs, which were analyzed by denaturing gel electrophoresis. R, right-hand side RNA fragment from reaction E experiment; L, left-hand side RNA fragment. nt, RNA size markers. The experiment was repeated three times with similar results. Source data are provided as a Source Data file.

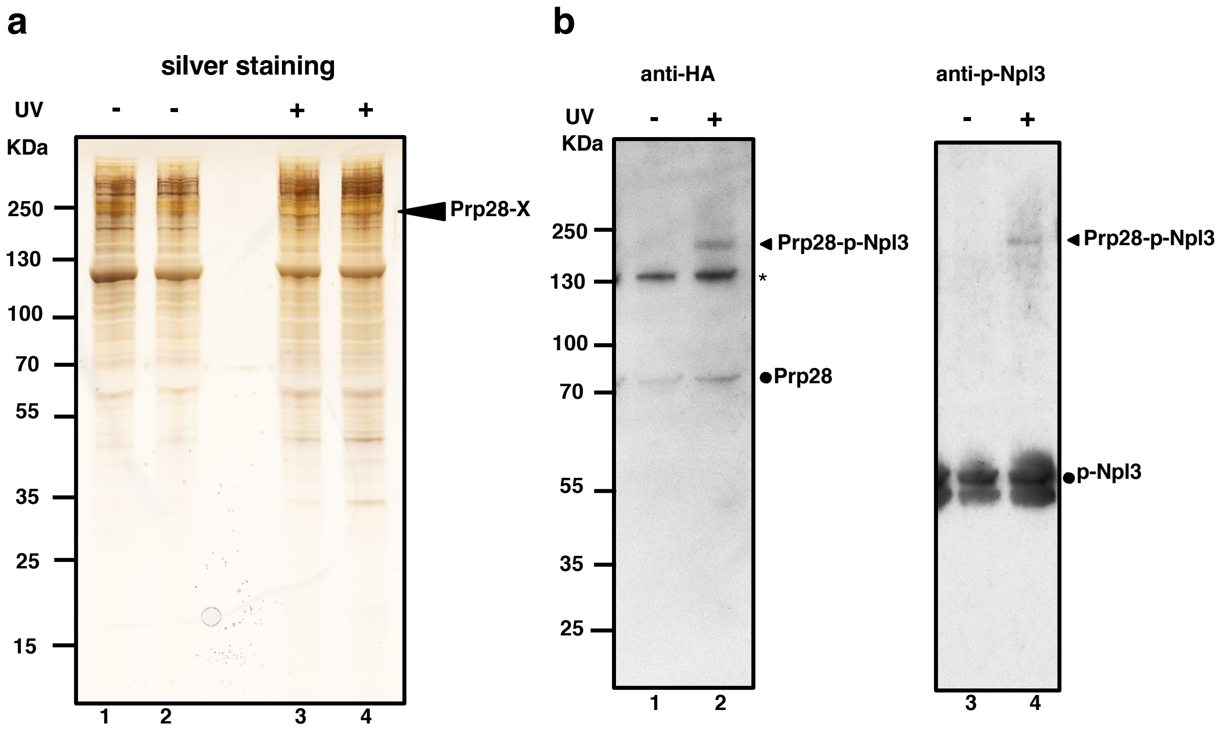

Supplementary Figure 8: Identification of the protein crosslinked to Prp28-E326^{BPA}. **a,** BPA-mediated crosslinking experiments were scaled up (100X). (-): without UV irradiation (lanes 1 and 2); (+) (lanes 3 and 4) with UV irradiation. Pulled-down materials were separated by 4–20% gradient SDS-PAGE and silver stained (lanes 1–4). Bands at this location (Prp28-X) from four individual lanes (-UV and +UV) were excised and then subjected to mass-spectrometry analysis. This experiment was repeated three times with similar results. **b,** The location of the crosslinked species were confirmed by immunoblotting. Validation of mass-spectrometry prediction of Npl3 as the crosslinked species. The left panel was probed with anti-HA antibody (lanes 1 and 2), and right panel with anti-p-Npl3 antibody (lanes 3 and 4), respectively. Asterisk, nonspecific background band. These experiments were repeated three times with similar results. Source data are provided as a Source Data file.

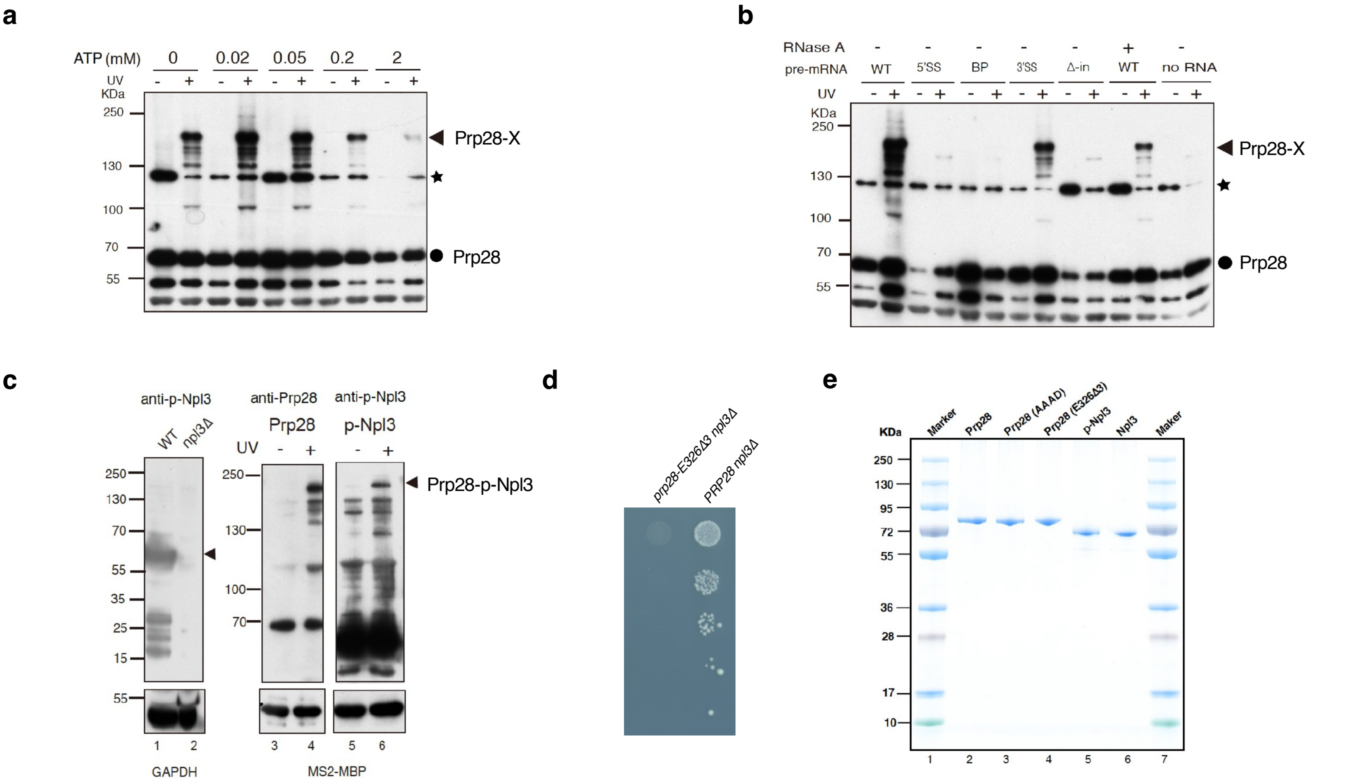

Supplementary Figure 9: Phosphorylated Npl3 is crosslinked to Prp28 in a splicing-dependent manner. **a-b,** Crosslink of Prp28-E326^{BPA} to protein X (triangle) correlates with optimal ATP conditions for pre-B complex formation. HA-tagged Prp28 and its crosslinked species were detected by anti-HA antibody. WT, wild-type transcript; 5'SS, 5'SS mutation (GUAUGU to AUUUGU); BP, branch-site A-to-C mutation (UACUAAC to UACUACC); 3'SS, 3'SS (AG|AG into AC| AC, where | indicates the 3' splice site); Δ-in, intron-less transcript. Star, nonspecific background band; filled circles, uncrosslinked Prp28. These experiments were repeated three times with similar results. **c,** Prp28-E326^{BPA} crosslinks to p-Npl3. Anti-p-Npl3 antibody detects Npl3 in wild-type extract, but not in *npl3Δ* extract (left panel). GAPDH, MS2-MBP, loading control. Anti-Prp28 and anti-p-Npl3 antibodies were used to probe the Prp28-E326^{BPA} crosslinked species to demonstrate the protein X as p-Npl3 (center and right panels). These experiments were repeated three times with similar results. **d,** Synthetic lethality caused by combining *prp28-E326Δ3* and *npl3Δ* mutations. **e,** Purified recombinant proteins (1 μg each) of Prp28, Prp28-AAAD mutant, Prp28-E326Δ3, p-Npl3, and Npl3 were separated by SDS-PAGE and stained by Coomassie Blue. Source data are provided as a Source Data file.

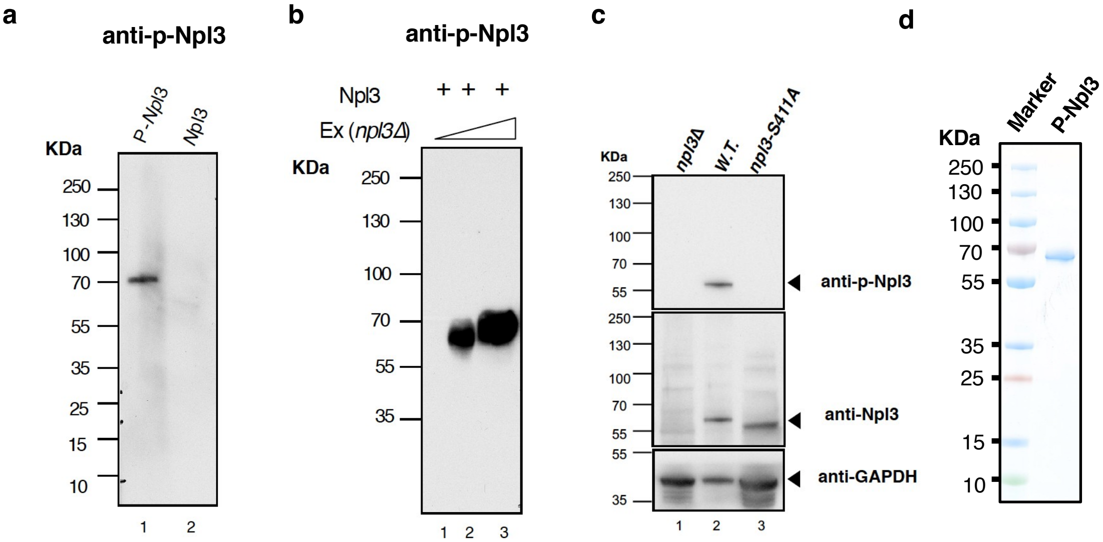

Supplementary Figure 10: Anti-p-Npl3 antibody specifically reacts with purified p-Npl3 but not Npl3. **a,** Immunoblotting of purified recombinant p-Npl3 and Npl3 proteins (80 ng in each lane) demonstrating that anti-p-Npl3 antibody does not cross-react with Npl3. **b,** Npl3 can be phosphorylated using extract (Ex) prepared from *npl3Δ3* strain. Purified Npl3 was mixed with increasing amount (0, 2, 10 μl) of extract (*npl3Δ*) in the presence of 1.8 mM ATP at 37˚C for 20 min and subjected to SDS-PAGE for immunoblotting analysis. **c,** Comparisons of immunoblotting of Ex (*npl3Δ*), Ex (W.T.), and Ex (*npl3-S411A*) showed that anti-p-Npl3 antibody specifically reacts with the epitope of Npl3-S411. **d,** Purified recombinant protein (1.2 μg) of p-Npl3 was resolved by SDS-PAGE and Coomassie Blue stained, then the single band was retrieved and analyzed for phosphorylation position by MS analysis. All above experiments were repeated three times with similar results. Source data are provided as a Source Data file.

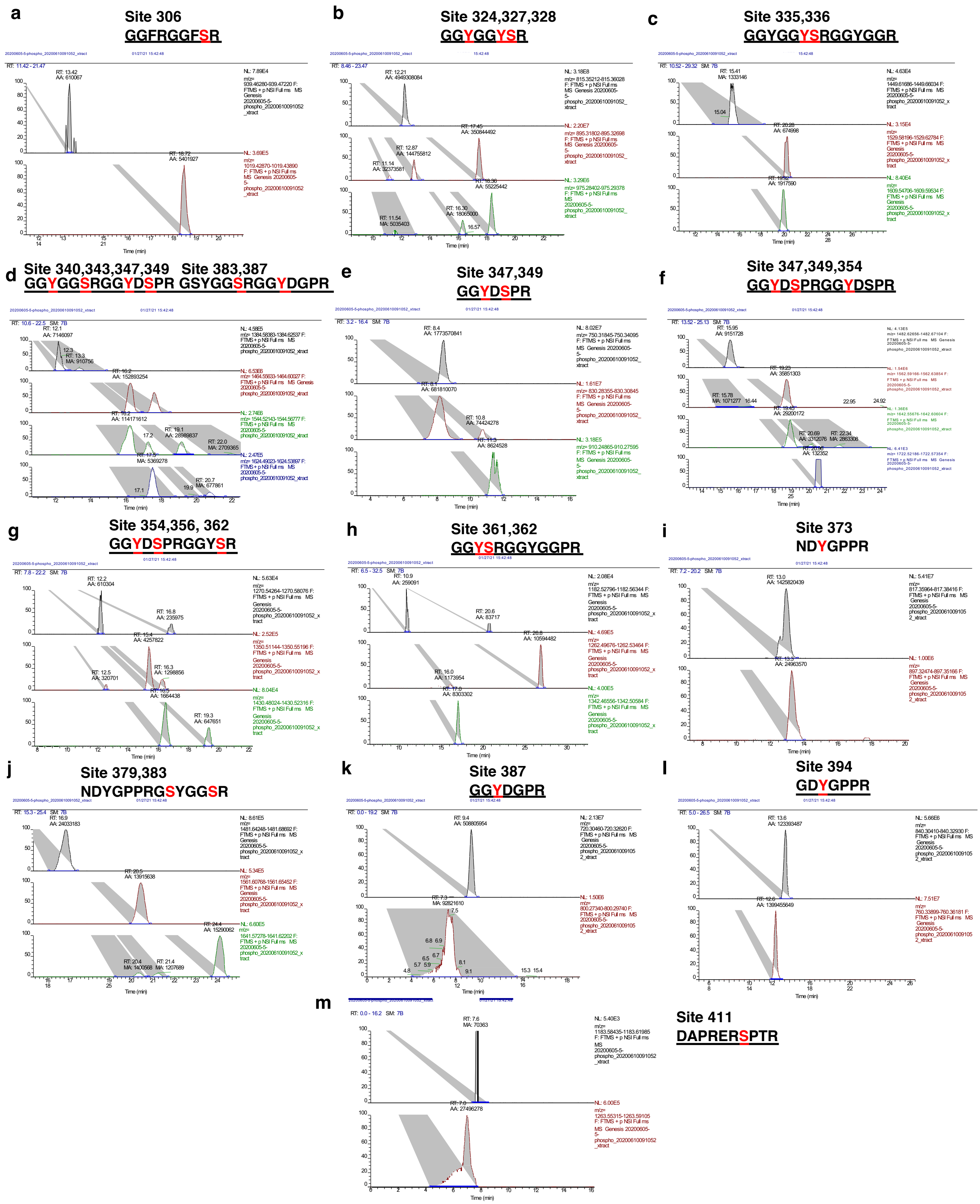

Supplementary Figure 11: LC-MS/MS Quantification of site-specific phosphorylation degree of p- Npl3 was done by Xcalibur software. **a.** site 306 **b.** site 324, 327, 328 **c.** site 335, 336 **d.** site 340, 343, 347, 349; site 383, 387 **e.** site 347, 349 **f.** site 347, 349, 354 **g.** site 354, 356, 362 **h.** site 361, 362 **i.** site 373 **j.** site 379, 383 **k.** site 387 **l.** site 394 **m.** site 411. The x-axis represents retention time (RT), The y-axis represents signal intensity or relative signal intensity. AA: automatic integration. MA: manual integration. Source data are provided as a Source Data file and PRIDE partner repository PXD024494.

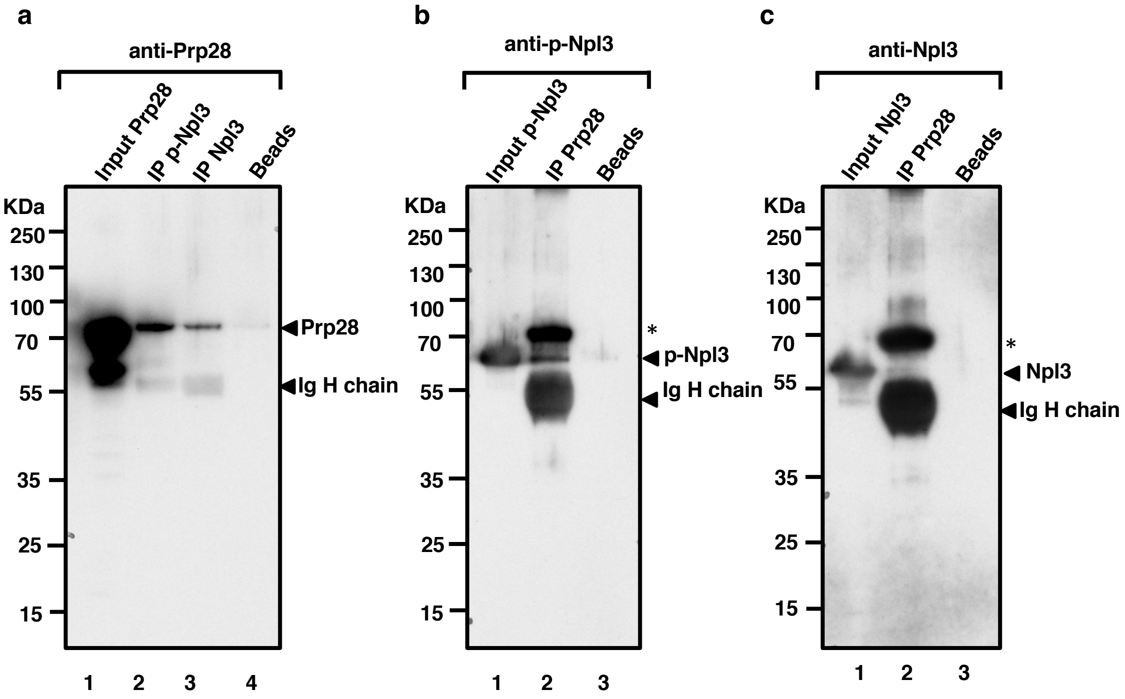

Supplementary Figure 12: Interactions of Phospho-Npl3 and Npl3 with Prp28. **a,** Anti-p-Npl3 antibody (recognizes only p-Npl3) and anti-Npl3 antibody (recognizes both p-Npl3 and Npl3) were pre-bound to Protein A beads. Prp28 was mixed either p-Npl3 or Npl3, preincubated, and then added to the antibody-bound Protein A beads. After incubation and washes, the bead-bound fractions were probed by Western analysis using anti-Prp28 antibody. The amount of Prp28 bound to p-Npl3 is apparently higher than that of the Npl3. "Beads" is a negative control without antibody. **b-c,** Reciprocal experiments of **a**, in which the bound antibody is anti-Prp28 antibody. The two experiments were probed by either anti-p-Npl3 or anti-Npl3 antibody, respectively, as shown on the top. Again, the amount of p-Npl3 bound to Prp28 is apparently higher than that of the Npl3. Asterisks in **b** and **c** are likely to represent a fraction of undenatured IgG heavy (Ig H chain) and light chains. All above experiments were repeated three times with similar results. Source data are provided as a Source Data file.

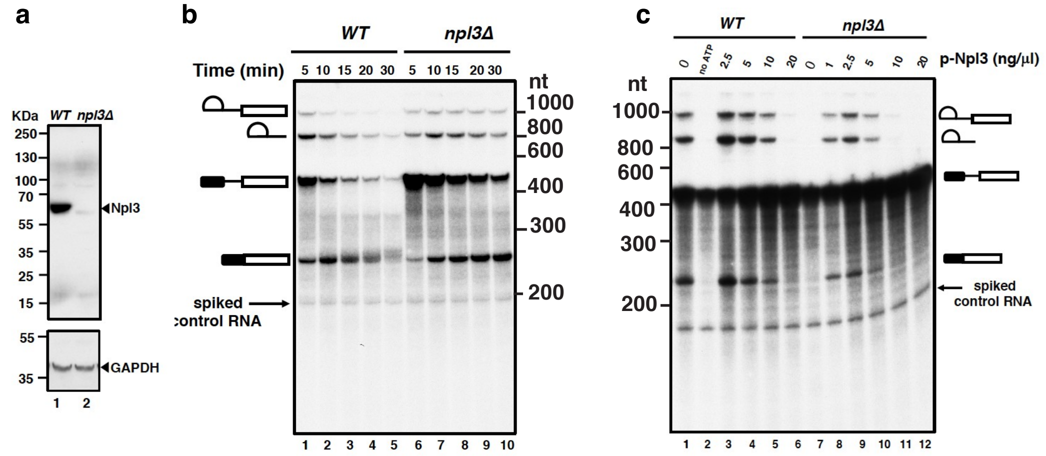

Supplementary Figure 13: Addition of p-Npl3 promotes splicing of Npl3-depleted extracts. **a,** Anti-Npl3 antibody detects Npl3 in the wild-type (WT) extract, but not in the *npl3Δ* extract. **b,** A time-course study of splicing reaction done with either *WT* or *npl3Δ* extracts. The rate of splicing in the *npl3Δ* extract is detectably slower than that of the wild type, which can be clearly seen from signals at 5 min (Cf. lanes 1 and 6). **c,** Dosage-dependent effect of adding p-Npl3 into the splicing reactions. p-Npl3 was added into either *WT* or *npl3Δ* splicing reaction and incubated for 5 min. The optimal dosage for improving *npl3Δ* splicing reaction is 2.5 ng/μl. Excessive amount of p-Npl3 appears to inhibit splicing for both types of reaction. A 200-nt hot transcript, used as a loading control, was spiked into the reaction when the reaction was completed. nt, RNA size markers. All above experiments were repeated three times with similar results. Source data are provided as a Source Data file.

Table S1: *brr2* alleles description

brr2 alleles	Amino acid changes	Phenotype
P3A	G1708 STOP	cold sensitive
P4A	L1971 STOP	cold sensitive
P5A	Q2037 STOP	cold sensitive
P6A	K2095 STOP	cold and temperature sensitive
P8A	Q1354K	cold sensitive
P9C	D1714V	cold sensitive
P10C	A1710T, T1794M	temperature sensitive
P12A	S1744R	temperature sensitive

* **All brr2 alleles are provided by Corina Maeder.**
