# Supplementary material for: Activation of Prp28 ATPase by phosphorylated Npl3 at a critical step of spliceosome remodeling
Source: Nat Commun. 2021 May 25;12:3082. doi: 10.1038/s41467-021-23459-4 (PMC8149812; doi:10.1038/s41467-021-23459-4)
Supplement: Supplementary file 12 — Description of Additional Supplementary Files [file 41467_2021_23459_MOESM12_ESM.pdf]

Description of additional supplementary information

Title: Supplementary Data 1

Description: Yeast strains used in this study.

Title: Supplementary Data 2

Description: Plasmids used in this study.

Title: Supplementary Data 3

Description: Oligonucleotides used in this study.

Title: Supplementary Data 4

Description: Summary of crosslinking Data.

Title: Supplementary Data 5a

Description: Mass spectrometry analysis of Prp28-K136BPA experiments

Title: Supplementary Data 5b

Description: Mass spectrometry analysis of Prp28-K136BPA experiments which related with RNA processing.

Title: Supplementary Data 6

Description: Summary of Prp8 genetic Data.

Title: Supplementary Data 7a

Description: Mass spectrometry analysis of Prp28-E326BPA experiments.

Title: Supplementary Data 7b

Description: Mass spectrometry analysis of Prp28-E326BPA experiments which related with RNA processing.

Title: Supplementary Data 8a

Description: LC-MS analysis of phospho (STY) probabilities of p-Npl3

Title: Supplementary Data 8b

Description: LC-MS Quantification of Site-specific phosphorylation degree of p-Npl3

Title: Supplementary Software 1

Description: The cryo-EM structure of the labeled human pre-B complex (Protein Data Bank 6QX9) was displayed by PyMol 2.3.2.
