# Supplementary material for: Activation of Prp28 ATPase by phosphorylated Npl3 at a critical step of spliceosome remodeling
Source: Nat Commun. 2021 May 25;12:3082. doi: 10.1038/s41467-021-23459-4 (PMC8149812; doi:10.1038/s41467-021-23459-4)
Supplement: Supplementary file 13 — Reporting Summary [file 41467_2021_23459_MOESM13_ESM.pdf]

nature research

Corresponding author(s): Tien-Hsien Chang

Last updated by author(s): Apr 21, 2021

# Reporting Summary

Nature Research wishes to improve the reproducibility of the work that we publish. This form provides structure for consistency and transparency in reporting. For further information on Nature Research policies, see our Editorial Policies and the Editorial Policy Checklist.

## Statistics

For all statistical analyses, confirm that the following items are present in the figure legend, table legend, main text, or Methods section.

| n/a | Confirmed | |
|---|---|---|
| ☐ | ☒ | The exact sample size (*n*) for each experimental group/condition, given as a discrete number and unit of measurement |
| ☐ | ☒ | A statement on whether measurements were taken from distinct samples or whether the same sample was measured repeatedly |
| ☐ | ☒ | The statistical test(s) used AND whether they are one- or two-sided<br>*Only common tests should be described solely by name; describe more complex techniques in the Methods section.* |
| ☒ | ☐ | A description of all covariates tested |
| ☒ | ☐ | A description of any assumptions or corrections, such as tests of normality and adjustment for multiple comparisons |
| ☐ | ☒ | A full description of the statistical parameters including central tendency (e.g. means) or other basic estimates (e.g. regression coefficient) AND variation (e.g. standard deviation) or associated estimates of uncertainty (e.g. confidence intervals) |
| ☐ | ☒ | For null hypothesis testing, the test statistic (e.g. $F$, $t$, $r$) with confidence intervals, effect sizes, degrees of freedom and $P$ value noted<br>*Give P values as exact values whenever suitable.* |
| ☒ | ☐ | For Bayesian analysis, information on the choice of priors and Markov chain Monte Carlo settings |
| ☒ | ☐ | For hierarchical and complex designs, identification of the appropriate level for tests and full reporting of outcomes |
| ☒ | ☐ | Estimates of effect sizes (e.g. Cohen's *d*, Pearson's *r*), indicating how they were calculated |

*Our web collection on statistics for biologists contains articles on many of the points above.*

## Software and code

Policy information about availability of computer code

| | |
|---|---|
| Data collection | Data collection is described in Methods.<br>(Protein and phospho peptide ID) Proteomics data acquisition: XcaliburTM 4.2.28.14 |
| Data analysis | - (Computational proteomics and phospho peptide ID): MaxQuant Version 1.6.14.0<br>- Graphs and statistical analysis: Graphpad Prism 8.0<br>- The Cryo-EM structure of the labeled human pre-B complex (Protein Data Bank 6QX9) is displayed by PyMol 2.3.2 and is stored as the file called Supplementary Software 1.<br>- RNA bands and the amount of ATP hydrolyzed were quantified and analyzed by TyphoonTM FLA9000 (GE healthcare Life Sciences) and ImageQuant TL7.0 (GE healthcare Life Sciences), respectively. |

For manuscripts utilizing custom algorithms or software that are central to the research but not yet described in published literature, software must be made available to editors and reviewers. We strongly encourage code deposition in a community repository (e.g. GitHub). See the Nature Research guidelines for submitting code & software for further information.

## Data

Policy information about availability of data

All manuscripts must include a data availability statement. This statement should provide the following information, where applicable:

- Accession codes, unique identifiers, or web links for publicly available datasets
- A list of figures that have associated raw data
- A description of any restrictions on data availability

The raw data from this study are available in the source file. The mass spectrometry proteomics data have been deposited to the ProteomeXchange Consortium via the PRIDE partner repository with the dataset identifier PXD024492 (Prp28-K136BPA), PXD024493 (Prp28-E326BPA), and PXD024494 (p-Npl3). The peptides ID of

PXD024492 and PXD024493 were analyzed by UniProt yeast database (UP000002311, 6049 entries). All other data supporting the findings of this study are available from the corresponding author on reasonable request.

# Field-specific reporting

Please select the one below that is the best fit for your research. If you are not sure, read the appropriate sections before making your selection.

☒ Life sciences ☐ Behavioural & social sciences ☐ Ecological, evolutionary & environmental sciences

For a reference copy of the document with all sections, see nature.com/documents/nr-reporting-summary-flat.pdf

# Life sciences study design

All studies must disclose on these points even when the disclosure is negative.

| | |
|---|---|
| Sample size | At least three biological replicates were used for each experimental condition. The design was based on prior assay experience and similar experiments reported in the literature. |
| Data exclusions | No data were excluded from the analyses. |
| Replication | All three biological replicates were successful. |
| Randomization | Samples were processed in random order. |
| Blinding | N/A |

# Reporting for specific materials, systems and methods

We require information from authors about some types of materials, experimental systems and methods used in many studies. Here, indicate whether each material, system or method listed is relevant to your study. If you are not sure if a list item applies to your research, read the appropriate section before selecting a response.

## Materials & experimental systems

| n/a | Involved in the study | |
|---|---|---|
| ☐ | ☒ | Antibodies |
| ☒ | ☐ | Eukaryotic cell lines |
| ☒ | ☐ | Palaeontology and archaeology |
| ☒ | ☐ | Animals and other organisms |
| ☒ | ☐ | Human research participants |
| ☒ | ☐ | Clinical data |
| ☒ | ☐ | Dual use research of concern |

## Methods

| n/a | Involved in the study | |
|---|---|---|
| ☒ | ☐ | ChIP-seq |
| ☒ | ☐ | Flow cytometry |
| ☒ | ☐ | MRI-based neuroimaging |

## Antibodies

| | |
|---|---|
| Antibodies used | Sources of the academic-lab produced antibodies used in this study are as follows: anti-Prp8 (Dr. Soo-Chen Cheng, Academia Sinica, Taiwan); anti-Snu114 (Dr. S.-C. Cheng, Academia Sinica, Taiwan); anti-Brr2 (Dr. S.-C. Cheng, Academia Sinica, Taiwan); anti-p-Npl3 and anti-Npl3 (Dr. Christine Guthrie, UCSF, USA); anti-Prp40 (Dr. Paul G. Siliciano, University of Minnesota, USA); anti-Prp28 (Dr. Tien-Hsien Chang, Academia Sinica, Taiwan).<br><br>Sources of the commercial antibodies are as follows: anti-HA.11 (ms, Cat # MMS-101R, Lot # B220850, Covance, Clone: 16B12); anti-V5-TAG (ms, Cat # MCA1360, Lot # 0915, Bio-Rad, Clone: SV5-PK1); anti-Maltose Binding Protein (MBP) (ms, Cat # E8032S, Lot # 0101603, NEB, Clone: B48); anti-GAPDH (ms, Cat # G8795-200, Lot # 045M4799V, Sigma); HRP-conjugated anti-rabbit IgG (H+L) (gt, Cat # 65-6120, Lot # QK229568, Invitrogen); HRP-conjugated anti-mouse IgG (H+L) (gt, Cat # 62-6520, Lot # QG215721, Invitrogen). Dilutions used for each antibody are described in Methods of the paper. |
| Validation | The validation that anti-p-Npl3 detects only p-Npl3 but not Npl3 and that anti-Npl3 detects both p-Npl3 and Npl3 was described in Siebel and Guthrie (1996). Validation of anti-Prp40 was described in Kao and Siliciano (1996). Validation of Anti-Prp28 was described in the Ph.D. Dissertation by Leah R. Stands (2003). Validation of anti-Prp8 and anti-Snu114 were described in Chung et al. (2019). Anti-Brr2 was raised against a His-tagged Brr2 N-terminal fragment (aa1-183) by Dr. Soo-Chen Cheng, Academia Sinica, Taiwan. This anti-Brr2 antibody detects, in a highly specific manner, Brr2 in splicing extract by both Western blotting and immunoprecipitation. Detailed citations of these antibodies can be found in the References of the main text.<br><br>Validation of commercial antibodies in specific species and applications can be found via links as follows.<br>V5 antibody: https://www.bio-rad-antibodies.com/monoclonal/viral-v5-tag-antibody-sv5-pk1-mca1360.html?f=purified#summary<br>HA antibody: https://www.biolegend.com/en-us/search-results/anti-ha-11-epitope-tag-antibody-11071<br>MBP antibody: https://international.neb.com/products/e8032-anti-mbp-monoclonal-antibody#Product%20Information |

GAPDH antibody: https://www.sigmaaldrich.com/catalog/product/sigma/g8795?lang=en&region=TW
Goat anti-Rabbit IgG (H+L) secondary antibody: https://www.thermofisher.com/antibody/product/Goat-anti-Rabbit-IgG-H-L-Secondary-Antibody-Polyclonal/65-6120
Goat anti-Mouse IgG (H+L) secondary antibody: https://www.thermofisher.com/antibody/product/Goat-anti-Mouse-IgG-H-L-Secondary-Antibody-Polyclonal/62-6520
